# Supplemental cardioplegia from direct left ventricle insertion for robotic mitral valve surgery in patients with aortic regurgitation

**DOI:** 10.1016/j.xjtc.2025.06.002

**Published:** 2025-06-18

**Authors:** Tomonari Uemura, Yasunari Hayashi, Toshikuni Yamamoto, Masato Mutsuga

**Affiliations:** Department of Cardiac Surgery, Nagoya University Graduate School of Medicine, Nagoya, Aichi, Japan

**Figure:**
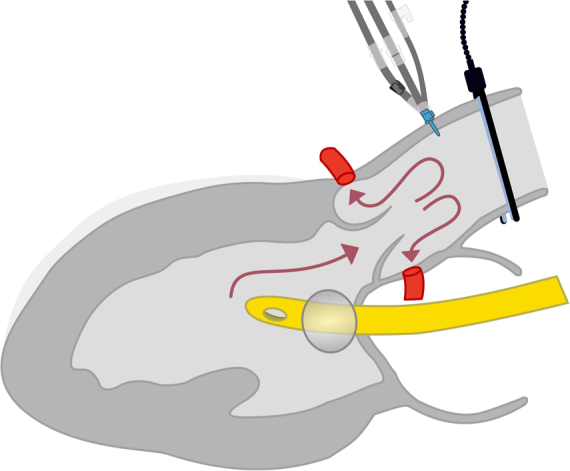
Direct left ventricular cardioplegia infusion during robotic mitral valve surgery in a patient with aortic regurgitation.

**Central Message**

Cardioplegia from direct left ventricular infusion offers a feasible technique for additional cardioplegia in robotic mitral valve surgery with aortic regurgitation instead of retrograde cardioplegia.

Minimally invasive cardiac surgery (MICS) presents challenges in surgical exposure. Because myocardial protection in MICS relies primarily on antegrade cardioplegia, greater than mild aortic valve regurgitation (AR) may affect the adequacy of antegrade cardioplegia delivery. Retrograde cardioplegia is another option, but the insertion takes valuable time during MICS surgery.^1^^,^^2^

We describe a simple technique for supplemental antegrade cardioplegia using direct left ventricle (LV) infusion in Da Vinci robotic-assisted mitral valve surgery with more than mild AR.

## Surgical Technique

This study was approved by the Nagoya University Hospital Ethics Committee (#2019-0179; approved August 23, 2019). Written informed consent for publication was obtained from all patients.

## Operative Setup

The left-hand port is positioned at the third intercostal space along the anterior axillary line, with the main port at the fourth intercostal space and the right-hand port at the fifth intercostal space. The endoscope is inserted through the main port, and an aortic cardioplegia needle with pressure monitor is inserted into the ascending aorta. After cross-clamping, antegrade cardioplegia (4:1 blood-crystalloid) is administered. If adequate pressure is not achieved, we proceed to the following method.

## Direct LV Cardioplegia Infusion

The technique is demonstrated in Video 1. The patient-side surgeon inserts a 24 Fr Foley catheter through the left atrial incision into the LV and inflates the balloon with sufficient saline (usually ≤20 mL) against the mitral annulus while maintaining careful monitoring to avoid subvalvular injury. Antegrade cardioplegia is then administered to fill the LV for deairing purposes. Following LV filling, additional cardioplegia is infused through the Foley catheter, while the console surgeon controls mitral regurgitation by passing the balloon through regurgitant areas or manipulating prolapsed leaflets with da Vinci forceps.

**Figure 1:**
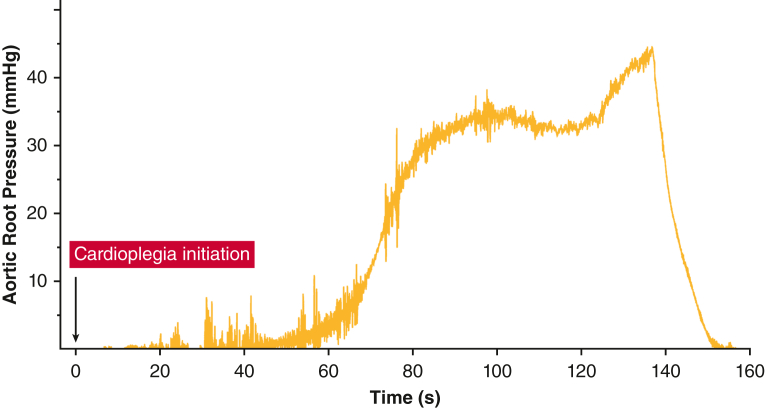
Aortic root pressure during cardioplegia delivery. Time course of aortic root pressure measured during direct left ventricular cardioplegia infusion, showing elevation to adequate coronary perfusion at 30 to 40 mm Hg.

**Figure 2:**
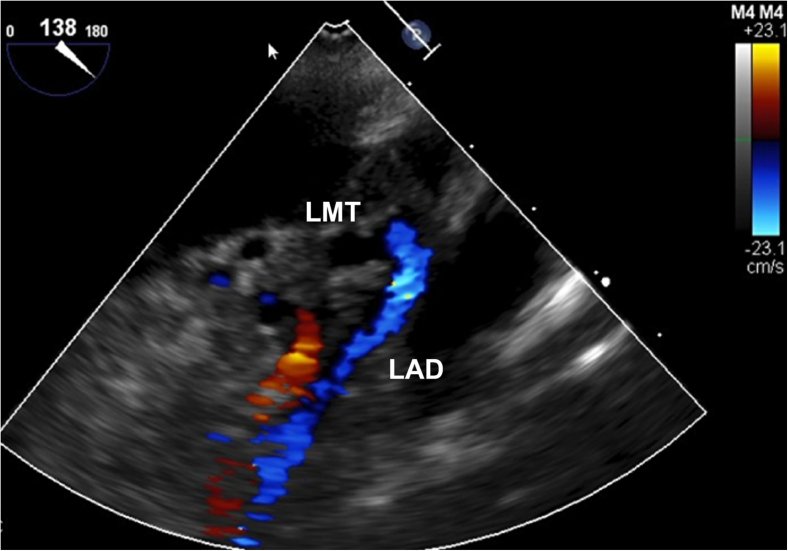
Doppler assessment of cardioplegia delivery. Transesophageal echocardiography with color Doppler shows cardioplegic flow from the left main trunk to the left anterior descending artery during direct left ventricular cardioplegia infusion.

We deliver cardioplegia at 100 to 140 mm Hg at a flow rate of 250 to 350 mL/minute. Adequate delivery is confirmed by an aortic root pressure increase to 30 to 40 mm Hg (Figure 1) and transesophageal echocardiography verification of flow through the left main coronary artery (Figure 2). Cardioplegia is administered in the same manner every 20 minutes. Before unclamping, hot shot cardioplegia is administered via the aortic root.

## Discussion

Ensuring adequate myocardial protection during MICS remains challenging in patients with AR. Retrograde cardioplegia can be used,^3^ but this requires additional superior vena cava and inferior vena cava isolation with atrial incisions, posing such risks as coronary sinus rupture, which may necessitate conversion to sternotomy.^1^^,^^2^

We have developed a technique for supplemental antegrade cardioplegia via direct LV infusion using the same surgical field. When the LV is filled and mitral regurgitation is controlled, the limited escape pathways direct excess cardioplegia into the coronary circulation. Pressure monitoring confirmed this hypothesis, and transesophageal echocardiography demonstrated effective perfusion of the left main trunk. The procedure is straightforward, requiring only a few additional minutes for catheter placement. In MICS, simplifying surgical techniques is considered preferable, and this method is highly rational.^4^

It could be argued that adequate myocardial protection can be achieved through antegrade cardioplegia alone if mitral regurgitation is properly controlled; however, our direct LV infusion technique using a Foley catheter offers several distinct advantages over this approach. First, the antegrade cardioplegia line uses a relatively narrow 14 G needle, which limits the flow rate. In contrast, the 24 Fr Foley catheter allows faster and more reliable delivery of cardioplegia at the same pressure. Second, in our target patients with mild to moderate AR, the regurgitant volume passing through the aortic valve is not substantial enough for efficient LV filling. With antegrade delivery alone, filling the LV would take longer and might create excessive pressure at the aortic root. Third, with our direct LV approach, the aortic valve remains open throughout the procedure, creating consistent pressure from the LV to the aortic root regardless of AR severity.

Three patients with more than mild AR who underwent this technique had postoperative cardiac biomarker levels comparable to those seen following our standard mitral valve procedures, with no evidence of unusual myocardial injury. In 1 patient, standard antegrade cardioplegia initially was deemed sufficient, but ventricular fibrillation developed. After supplemental LV infusion, cardiac arrest was rapidly reestablished, demonstrating the effectiveness of this technique.

Although LV distention during cardioplegia delivery is a potential concern, pericardial traction sutures in MICS effectively constrains lateral and posterior ventricular expansion, mitigating the risk of excessive distention. This concept is supported by a report on water tests in mitral valve repair, where controlled aortic root pressure (≤60 mm Hg) during LV pressurization was deemed safe.^5^ Our technique produced aortic root pressures ≤40 mm Hg, suggesting an even greater safety margin.

## Conclusions

Our technique of supplemental cardioplegia from direct LV insertion provides safe and effective myocardial protection in robotic mitral valve surgery with more than mild AR, offering a practical alternative to retrograde cardioplegia without additional atrial incisions or increased procedural complexity.

## Conflict of Interest Statement

The authors reported no conflicts of interest.

The *Journal* policy requires editors and reviewers to disclose conflicts of interest and to decline handling or reviewing manuscripts for which they may have a conflict of interest. The editors and reviewers of this article have no conflicts of interest.

## Supplementary Data

Video 1 Direct left ventricular supplemental cardioplegia technique for robotic mitral valve surgery. Video available at: https://www.jtcvs.org/article/S2666-2507(25)00251-2/fulltext.
