# Supplementary figures and images for: Supplemental cardioplegia from direct left ventricle insertion for robotic mitral valve surgery in patients with aortic regurgitation
Source: JTCVS Tech. 2025 Jun 18;33:79–81. doi: 10.1016/j.xjtc.2025.06.002 (PMC12529712; doi:10.1016/j.xjtc.2025.06.002)

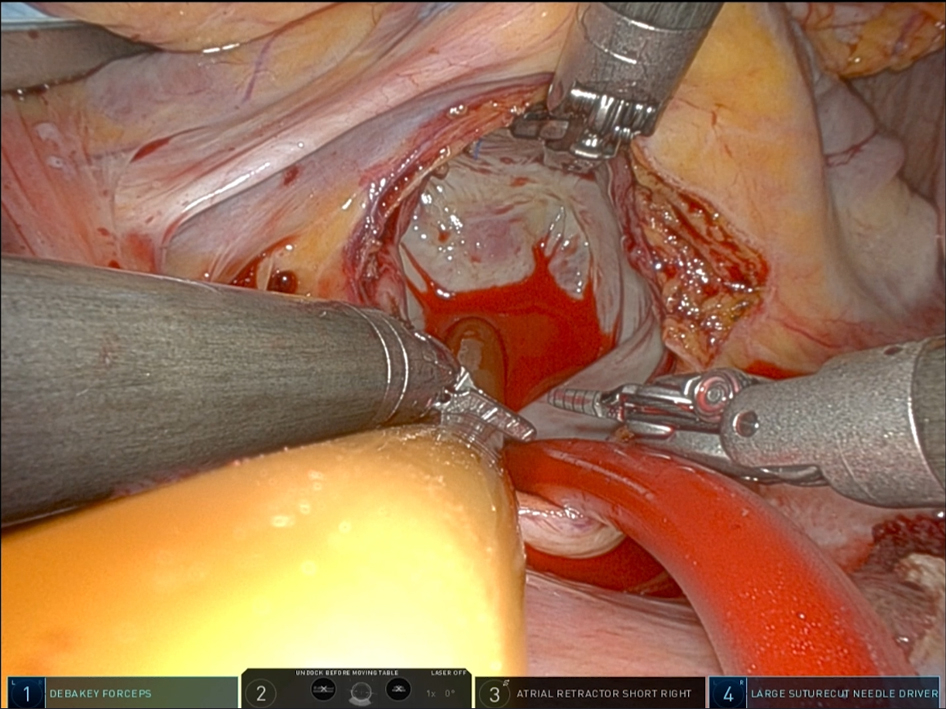

Supplement: Video 1 — Direct left ventricular supplemental cardioplegia technique for robotic mitral valve surgery. Video available at: https://www.jtcvs.org/article/S2666-2507(25)00251-2/fulltext. [file fx2.jpg]
